# Clinical spectrum of contactin-associated protein 2 autoimmune encephalitis in children

**DOI:** 10.3389/fnins.2023.1106214

**Published:** 2023-05-18

**Authors:** Wenjing Hu, Enhui Wang, Hongjun Fang, Li Li, Jurong Yi, Qingqing Liu, Wei Qing, Danni Guo, Qianqian Tan, Hongmei Liao

**Affiliations:** ^1^Department of Neurology, Hunan Children’s Hospital, Changsha, Hunan, China; ^2^Department of Radiology, Hunan Children’s Hospital, Changsha, Hunan, China

**Keywords:** CASPR2, autoimmune encephalitis, children, retrospective assessment, overlap syndrome

## Abstract

**Objective:**

Anti-contactin-associated protein 2 (CASPR2)-related autoimmune encephalitis (AE) is more common in adults than in children. Clinical understanding of anti-CASPR2-antibody (Ab)-related AE, diagnosis and treatment standards are lacking in children. Therefore, this retrospective study on clinical symptoms and treatment outcomes in children with anti-CASPR2-Ab-related AE was conducted, to improve the clinical understanding of the disease, its diagnosis and treatment.

**Methods:**

This study retrospectively assessed children with anti-CASPR2-Ab-related AE from January 1, 2020, to June 30, 2022, in the Department of Neurology at Hunan Children’s Hospital. Data regarding demographics, clinical symptoms, laboratory examinations, electroencephalography (EEG), imaging, and curative were collected.

**Results:**

Thirteen patients were positive for serum anti-CASPR2-Ab (age at manifestation, 25 months to 13 years old; median, 8.1 years old; male-to-female ratio, 8/5). One patient (P1) had dual Abs, including anti-CASPR2 and anti-N-methyl-D-aspartate receptor Abs; his symptoms were more severe than those of children with anti-CASPR2 Abs alone. The clinical symptoms of the 13 patients with anti-CASPR2 Ab were movement disorders (9/13), consciousness disorders (9/13), abnormal demeanor (8/13), seizures (7/13), language disorders (6/13), fever (6/13), pain (4/13), involuntary exercise (4/13), poor diet (4/13), vomiting (3/13), sleep disorders (3/13), mood disorders (3/13), eczema/itching/redness (2/13), sweating (P8), urinary disorders (P13), and cognitive disorders (P9). No tumors were found in any patient. Additionally, EEG results of six patients were abnormal and imaging findings such as abnormal signals were found in 10 patients. Moreover, all except one patient recovered well after treatment; P1 with overlapping syndrome underwent recovery for more than 2 years. None of the patients who recovered have had a relapse.

**Discussion and conclusion:**

Anti-CASPR2-Ab-related AE has several clinical manifestations. Anti-CASPR2-Ab levels were higher in male patients than in female patients. Moreover, related tumors are relatively rare. Most patients benefit from immunotherapy and have a lower chance of recurrence in the short term. Furthermore, different from patients who had anti-CASPR2-Ab AE alone, those with overlapping syndrome had a severe and complex condition requiring lengthy treatment and rehabilitation. Additional studies are needed to evaluate the long-term prognosis of these patients.

## 1. Introduction

Autoimmune encephalitis (AE) is an inflammatory disease of the central nervous system, clinically characterized by altered consciousness and/or abnormal behavior. And it can also occur secondary to a central nervous system infection or tumors that triggering an immune response (Brenton and Goodk, 2016; Cellucci et al., 2020). Currently, the prevalence of AE is 10–20% in encephalitis cases (Dutra et al., 2018; Hermetter et al., 2018); anti-N-methyl-D-aspartate receptor (NMDAR)-antibody (Ab)-related encephalitis is the most common, followed by anti-leucine-rich glial inactive protein 1 (LGI1), anti-γ-Gammaaminobutyric acid type B receptor (GABABR) and anti-contactin-associated protein-like2 (CASPR2)-Ab-related encephalitis. The CASPR2 protein is widely expressed in the central and peripheral nervous system neurons (Saint-Martin et al., 2018). Anti-CASPR2-Ab-related AE has complex and diverse symptoms, such as limbic encephalitis, peripheral nerve hyperexcitability, Morvan syndrome, and cerebellar syndrome (Boyko et al., 2020). Multiple centers have detailed anti-CASPR2-Ab-related AEs in adult patients (Qin et al., 2021). Currently, anti-CASPR2-Ab-related AE in children is relatively rare in clinical practice (López-Chiriboga et al., 2018). The clinical understanding of the disease, its diagnosis, treatment procedures, and standards are lacking. Thus, clinical diagnostic methods are challenging. The present study retrospectively analyzed the general data and clinical characteristics, laboratory and imaging examination results, treatment, and prognosis of patients with anti-CASPR2-Ab-related AE to improve the clinical understanding of the disease, its clinical diagnosis, and treatment.

## 2. Materials and methods

### 2.1. Patients

We retrospectively collected the clinical data of 13 children who were hospitalized at Hunan Children’s Hospital in China, between January 1, 2020, and June 30, 2022, and tested positive for anti-CASPR2-Ab. The inclusion criteria were as follows:

1. Patients with neurological or psychiatric symptoms, or both, in a previously healthy child within 3 months.
2. Patients with two or more of the following clinical manifestations: changes in mental status/level, slowed consciousness, electroencephalography (EEG), or epileptiform activity, focal neurological deficits, cognitive dysfunction, movement disorders, psychiatric symptoms, seizures limited to the previously known seizure episodes or other conditions with two or more features.
3. Patients with magnetic resonance images (MRI) and EEG features of encephalitis, excluding other neurological disorders.
4. Patients with AE-related autoantibodies in serum or cerebrospinal fluid (CSF) samples or both.
5. Reasonable exclusion of alternative causes.

Based on the clinical history and necessary auxiliary examinations, other diagnoses, such as genetic and metabolic diseases, were excluded. The guardians of all participants recruited for this study provided written informed consent before their participating. The Ethics Committee of Hunan Children’s Hospital approved the study (NO. KS2021-65). Our cohort enrolled 13 patients with anti-CASPR2-Ab-associated encephalitis.

### 2.2. Laboratory tests

The diagnosis was confirmed using detailed clinical examination, imaging, and blood and CSF examinations. Serum and CSF autoimmune neuronal Ab panels were routinely tested for each patient suspected of having AE. The spectrum of AE-related-Abs were tested included the anti-NMDAR, anti-LGI1, anti-glutamic acid decarboxylase (GAD65), anti-CASPR2, anti-α-amino-3-hydroxy-5-methyl-4-isoxazolepropionic acid receptor (AMPAR), and anti-γ-aminobutyric acid type B (GABAB) Abs. The serum and CSF samples that tested positive for AE-related-Abs were evaluated using a cell-based assay (CBA) in the laboratory (Wuhan Kindstar Medical Laboratory Co., LTD., Wuhan, China). Samples of patients tested positive for anti-CASPR2-Ab were mixed with a dilution of Abs (1:10, 1:32, 1:100, 1:320). When incubated with the patient samples, the panel slides were coated with fixed cells overexpressing the IgG-binding self-antigen associated with AE; this antigen bound to the specific Abs. After incubation with Alexa Fluor 594-labeled goat anti-human IgG (Invivogen, California, USA), slides were observed under a fluorescence microscope. Furthermore, we conducted tissue-based assay (TBA) fluorescence staining on some samples, which combined the anti-nerve Abs in the patients’ serum or CSF with the monkey brain cells to form a specific fluorescence. The indirect immunofluorescence protocol was followed the manufacturer’s recommendations (Invivogen, California, USA).

We evaluated immunoglobulin (Ig) Abs and serum complement levels in detail. Blood biochemical indices, electrolyte levels, eight thyroid function items, liver and kidney functions, and myocardial-related indicators were also tested. Additionally, we performed biochemistry and staining (bacteria, tuberculosis, and fungi) and whole-blood lymphocyte subgroup detection in CSF on the children exclude infection. Finally, patients with unexplained psychiatric symptoms were also tested for AE-related Abs.

Patients were required to complete the first EEG examination within 1 week of admission after the first clinical symptoms, using a paperless EEG-1200C (Nippon Optoelectronics) EEG instrument. Two certified EEGs technicians recorded all EEGs using uniform case notes. MRI was performed using a Philips NT-3.0 T scanner (Eindhoven, Netherlands). T1 weighted imaging (T1WI), T2 weighted imaging (T1WI), and fluid-attenuated inversion recovery (FLAIR) imaging of the head and spinal cord were performed for all patients.

### 2.3. Modified rankin scale score

The modified Rankin Scale (mRS) score was used to retrospectively evaluate the severity of neurology at onset and the results of the final follow-up (Zhang et al., 2017). Follow-up was conducted throughout patient visits or telephone interviews.

The good long-term prognosis was defined as an mRS score ≤ 2, and the poor long-term prognosis was defined as an mRS score > 2. Relapse was defined as the recurrence of symptoms after a full or partial recovery, with at least 2 months of sustained improvement (van Sonderen et al., 2016).

### 2.4. Statistical analysis

SPSS 19.0 was used for statistical analysis. Descriptive statistics were applied to analyze clinical data, such as medians.

## 3. Results

**Figure 1:**
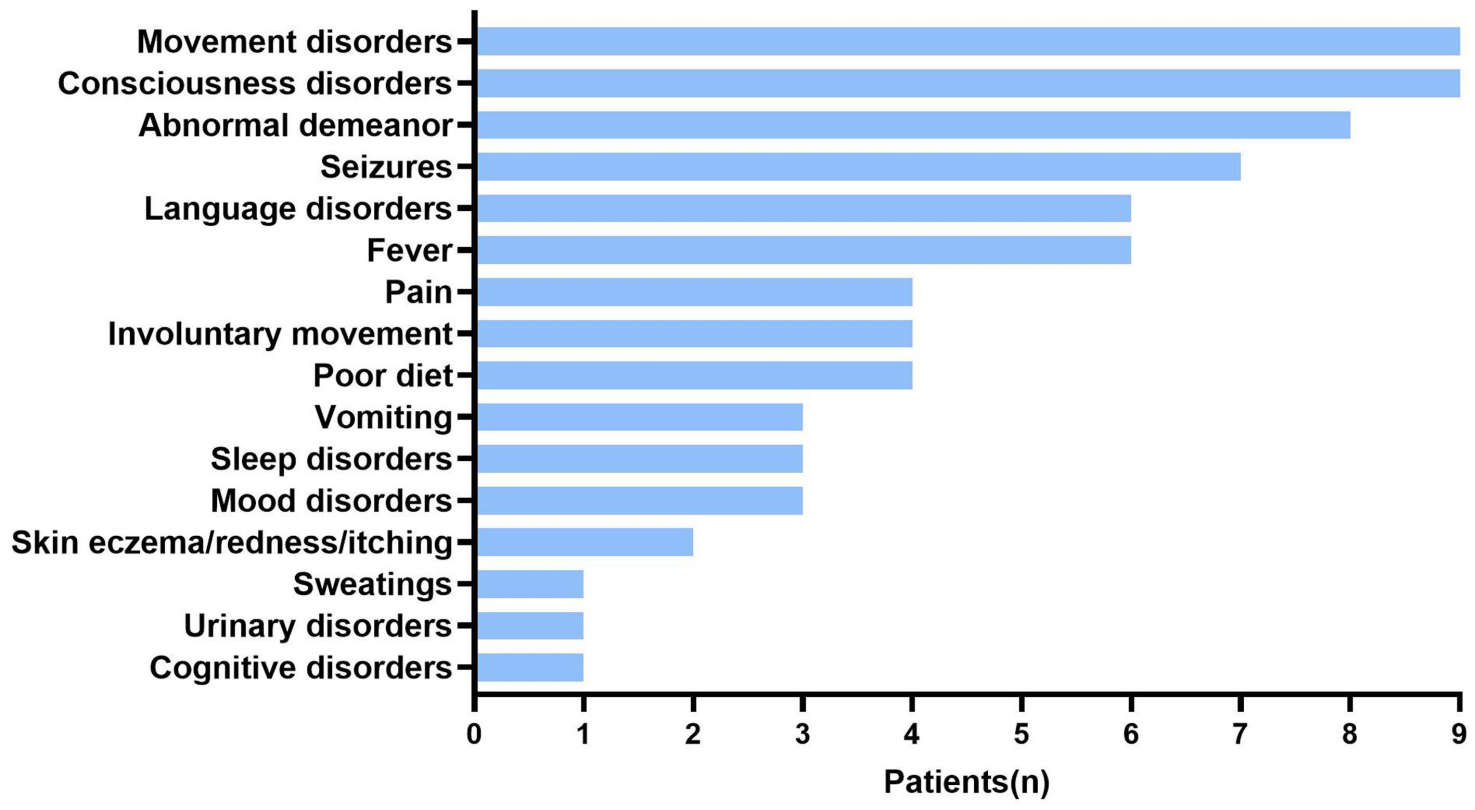
Clinical symptoms in patients with anti-CASPR2 Ab. Among 13 patients, clinical symptoms include movement disorders (9/13), consciousness disorders (9/13), abnormal demeanor (8/13), seizures (7/13), language disorders (6/13), fever (6/13), pain (4/13), involuntary movement (4/13), poor diet (4/13), vomiting (3/13), sleep disorders (3/13), mood disorders (3/13), skin eczema (2/13), sweating (1/13), urinary disorders (1/13), and cognitive disorders (1/13). CASPR2, Contactin-Associated Protein-like 2; Ab, antibody.

Thirteen children had serum anti-CASPR2-Ab titers ≥1:10 among 177 children with suspected of having AE under 18 years old. Based on clinical features, imaging and laboratory data, 13 patients (8:5, male: female ratio) positive for anti-CASPR2-Ab were finally included in this study. No tumors were found in any patient. Figure 1 and Table 1 show the detailed demographic and clinical characteristics of these patients with anti-CASPR2-Ab-related AE.

**Table 1:** Clinical characteristics of children with anti-CASPR2 Ab.

NO.	Age at disease onset (y)/sex	Clinical diagnosis	Clinical manifestation	EEG	Neuroimaging findings
P1	9/male	Morvan syndrome/epilepsy syndrome/pain syndrome	Abnormal psychological and behavioralities, unprovoked crying, restlessness, fidgeting, fever, poor diet, seizures, cramps, no response, involuntary movement, skin eczma, leg pain, stomachache	1.Background rhythm slowing; 2.Continuous rhythmic distribution of sharp wave and sharp slow wave in the left frontal and temporal (frontal) area; 3.Continuous focal discharges.	Multiple spottyandpatchy abnormal signals (and blurred edges) in the bilateral frontal parietal lobe white matter, hyperintensity on fluid-attenuated inversion recovery images.
P2	8.1/male	Epilepsy syndrome	Unprovoked crying, seizures, fever	1.Increased slow waves in the posterior head on the left side and poor rhythm in the left occipital region; 2.Sharp waves and sharp slow waves distributed in the left occipital area.	Long T2 signal strip in the left parietto-occipital cortex, pressure water image showed high signal and slightly thick local cortex.
P3	8.3/male	Anti-CASPR2-Ab-related AE	Abnormal psychological and behavioralities	Normal	Slightly enlarged and deepened bilateral hemispheric sulcus fissure.
P4	13.8/female	Anti-CASPR2-Ab-related AE	Unsteady gait, ataxia, headache	Normal	1.Arachnoid cyst in posterior cranial fossa; 2.Bilateral lateral ventricles slightly plump.
P5	5.1/male	Anti-CASPR2-Ab-related AE	Abnormal psychological and behavioralities, no response	Normal	Normal
P6	12.6/female	Anti-CASPR2-Ab-related AE/Japanese encephalitis	Abnormal psychological and behavioralities, cramps, seizures, no response, vomiting, poor diet, fever, headache	Normal	1.Bilateral signal abnormalities in the basal thalamic ganglia; 2.Enlarged sulcus fissure in the bilateral hemisphere and slightly longer T2 signal appeared in the white matter of the bilateral frontal lobe.
P7	6.5/male	Anti-CASPR2-Ab-related AE	Seizures, cramps, no response	Small quantities of spike waves in the left frontal pole and frontal region.	Abnormal signal shadows in bilateral frontal and left temporal cortex and subcortical cortex.
P8	2.1/female	Anti-CASPR2-Ab-related AE	Abnormal psychological and behavioralities, seizures, vomiting, cramps, sleep disorders, weakness of limbs, unsteady gait, skin eczema	Normal	Widened extracerebral space of left temporal.
P9	9.8/male	Limbic encephalitis/Morvan syndrome	Abnormal psychological and behavioralities, restlessness, unconsciousness, mood disorders, seizures, no response, involuntary movement, sleep disorders, movement disorders, cognitive disorders	Normal	Abnormal signal shadow in the bilateral thalamus and right hippocampus.
P10	4.9/male	Anti-CASPR2-Ab-related AE	Seizures, cramps, no response, salivation, involuntary movement, ataxia, fever	Slow background, a large number of δ rhythms in the frontal polar, frontal and anterior temporal regions.	Multiple abnormal signals in the bilateral caudate head and bilateral frontotemporal parietal occipital cortex.
P11	7.4/male	Limbic encephalitis/Morvan syndrome/epilepsy/pain syndrome	Seizures, cramps, involuntary movement, sleep disorders, headache, leg pain, fell suddenly	1.Occipital area rhythm slowing; 2.Sharp wave in the right frontal area; 3.Focal episodes in a persistent state.	Normal
P12	9.1/female	Anti-CASPR2-Ab-related AE	Abnormal psychological and behavioralities, intelligence and attention reduce, poor memory, poor diet, fever, dizziness, hyperhidrosis	Background rhythm slowing.	Normal
P13	6.8/male	Limbic encephalitis/Morvan syndrome	Unresponsive, slurred speech, language reduction, uracratia, vomiting, poor diet, fever, ataxia	Normal	Bilateral thalamus and basal ganglia punctate abnormal signal, enhanced part of the sulcus fissure bar enhancement shadow.

NO, number; P, patient; y, years; F, female; M, male; EEG, electroencephalogram; MRI, magnetic resonance imaging; CASPR2, contactin-associated protein-like 2; FLAIR, fluid attenuated inversion recovery.

### 3.1. Demographic features and clinical manifestations

Among the 13 patients, the age of symptom onset ranged from 25 months to 14 years old, with a median age of 8.1 years old. Eight patients had psycho behavioral disorders, including abnormal psychological and behavior (6/13) as well as unprovoked crying (2/13). Eight patients had seizures, which manifested as eyes turning up, eyes staring, and oral cyanosis (7/13), no response (7/13), and one patient (P9) was initially restless but later became unconscious to the outside world. Additionally, P12 had decreased attention, and P13 had slurred speech due to extrapyramidal lesions during the disease and 3/13 patients developed varying degrees of sleep disorders (Figure 1; Table 1).

**Figure 2:**
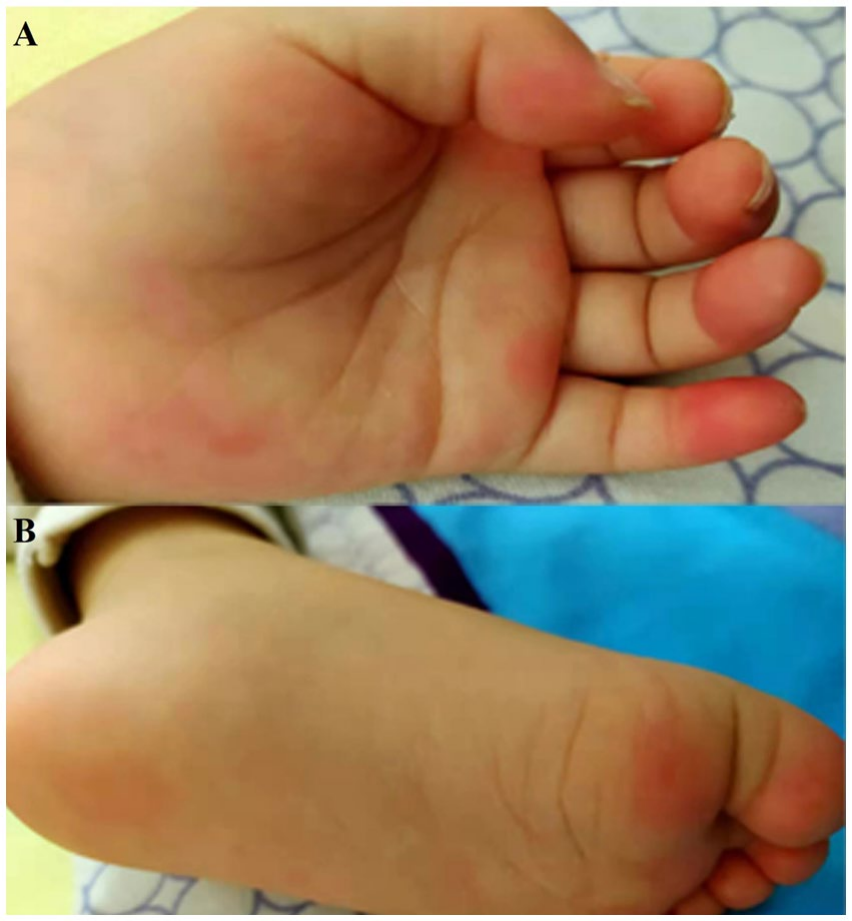
Clinical symptoms in patient with palmoplantar erythema. There were red spots and red rash on hands **(A)** and feet **(B)** of patient 8.

The symptoms of autonomic dysregulation included ramps (6/13), fever (6/13), involuntary movements (4/13), skin eczema and redness (2/13), poor diet (3/13), vomiting (3/13), urinary incontinence (P13), and sweating (P12) (Table 1). One girl (P8) developed observable palmoplantar erythema (Figure 2).

Neuromuscular symptoms were present in half of the cases. Abnormal gait (3/13), limb weakness (P8), sudden fall (P11), and ataxia (3/13) were also noted (Figure 1; Table 1).

### 3.2. Analysis of AE Abs using CBA and TBA

**Figure 3:**
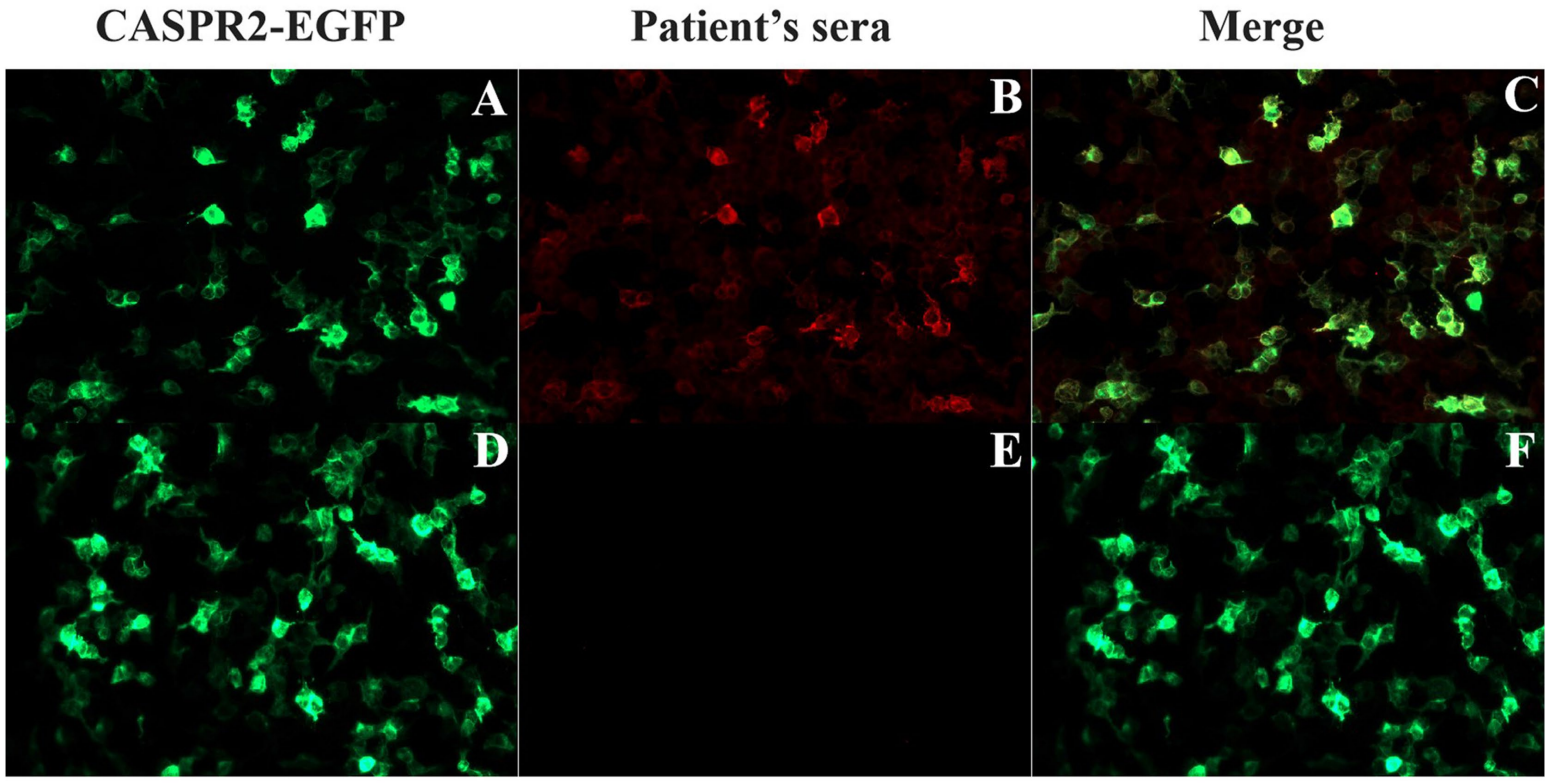
Detection of anti-CASPR2 Ab by CBA in patients. **(A–C)** HEK293T cells transfected with EGFP-labeled CASPR2 plasmid that co-express CASPR2 and EGFP, show reactivity with sera from the P9 with autoimmune encephalitis in acute exacerbation period. **(D–F)** Serum collected from P9 two years after initial onset showing negative staining. CASPR2, Contactin-Associated Protein-like 2; Ab, antibody.

All patients were tested for six kinds of serum Abs about AE (anti-NMDAR, anti-LGI1, anti-GAD65, anti-CASPR2, anti-AMPAR and anti-GABABR) using the CBA method and all were positive for anti-CASPR2-Ab. In the patients who had fully recovered, the Ab titer evaluation were yielded negative results. Figure 3 shows the anti-CASPR2-Ab CBA serum double fluorescence staining results of P9. Moreover, a CSF test for the six Abs was performed in seven patients, showing Ab titers of 1:10 in four patients (4/7) and 1:1 in two patients (2/7), with negative result in one patient (P8). Additionally, P1 had anti-NMDAR-Ab in his CSF sample at a 1:32 Ab titer, but the serum anti-NMDAR-Ab result was negative. Moreover, five patients completed the TBA test, and the results were negative in one patient (P2) and positive in four patients (4/5). Supplementary Figure S1 shows the results of TBA monkey brain staining for P9.

### 3.3. Biochemical analysis

Abnormal results in blood biochemical indices included elevated levels of lactate dehydrogenase (P10), lactate (4/13), and blood glucose (3/13). P1 had an electrolyte imbalance and low potassium ion levels. Additionally, biochemical tests in the CSF (12/13) revealed increased total cell count (5/12), leukocytosis (2/12), elevated protein (2/12), elevated lactate dehydrogenase levels (3/12), and a significant increase in lactate dehydrogenase levels in one girl (P10). The chlorine ion levels were abnormal in five patients (5/12), indicating a slight increase (Table 2).

**Table 2:** Treatment and follow-up of patients with positive anti-CASPR2 Ab.

NO.	Anti-CASPR2-abtiter in serum 1:n	Anti-CASPR2-abtiter in CSF 1:n	Immune therapy (response and time after disease onset)	Maintenance therapy and supportive treatment	mRS of onset	mRS at the last follow-up	Outcome	Additional notes
P1	1:10	/	IVIG, IVMP (no improvement)	Oral Prednisone (less improvement)	4	2	Poor memory, poor computing ability, Poor completion of fine movements and slurred words	CSF: elevated lactate dehydrogenase; Blood: isolated fT4 elevation, increased creatine kinase and myoglobin, IgE\IgG\IgM elevation, low WBC, Hb and PLT, decreased potassium
P2	1:10	1:10	/	Carbamazepine (improvement)	2	1	Complete remission	Blood: slightly elevated blood lactate
P3	1:10	/	IVIG, IVMP (improvement)	Oral Prednisone (improvement)	3	0	Complete remission	CSF: chlorine elevation
P4	1:10	/	IVIG, IVMP (improvement)	Oral Prednisone (improvement)	3	0	Complete remission	CSF: increased WBC, protein and chlorine, lactic acid; Blood: amylaceum and chlorine elevation
P5	1:10	/	/	/	3	1	Complete remission	Blood: elevated amylaceum and purine trione
P6	1:10	/	IVIG, IVMP (less improvement)	Oral Prednisone (improvement)	4	1	Complete remission	CSF: slightly increased total cell count, mild protein elevation; Blood: elevated WBC and IgG/IgM, decreased C3 and C4
P7	1:10	/	IVIG, IVMP (improvement)	Oral Prednisone (improvement)	3	0	Complete remission	Blood: increased WBC, a slight increase amylaceum
P8	1:100	negative	IVIG, IVMP (improvement)	/	2	0	Complete remission	CSF: a slight increase chlorine; Blood: lactic acid and creatine kinase isoenzymes elevation
P9	1:100	1:10	IVIG, IVMP, RTX (no improvement)	Oral Prednisone, Clonazepam, Carbamazepine (improvement)	3	1	Complete remission	CSF: a slight increase chlorine; Blood: elevated WBC, PLT and ALT; IgA/IgE/IgG/IgM, B lymphocytes and C4 elevation
P10	1:10	1:10	IVIG (improvement)	/	2	0	Complete remission	CSF: significantly elevated lactate dehydrogenase and elevated chlorine; Blood: lactate dehydrogenase, AST and PLT elevation
P11	1:10	1:10	/	Oxcarbazepine (improvement)	3	1	Complete remission	Blood: total bile acid and C4 elevation
P12	1:10	/	/	Proton pump inhibitor, Amoxicillin, Clarithromycin (improvement)	2	1	Complete remission	Blood: Low C3
P13	1:10	1:1	IVIG, IVMP (improvement)	Oral Prednisone, Acyclovir (improvement)	3	0	Complete remission	CSF: elevated lactate dehydrogenase and WBC; Blood: IgA\IgE\IgG\IgM, B lymphocytes and blood lactate elevation, decreased T lymphocytes

NO, number; P, patient; mRS, modified Rankin Scale; IVIG, intravenous immunoglobulins; IVMP, intravenous methylprednisolone; RTX, rituximab; CSF, cerebrospinalfluid; /, No relevant testing or treatment; WBC, White Blood Cell Count; Hb, Hemoglobin; PLT, platelets transfusion; ALT, alanine aminotransferase; AST, aspartate aminotransferase; C3, complement 3; C4, complement 4.

### 3.4. Additional laboratory tests

Four patients had abnormal liver and kidney function indices, including increased total bile acid (P12), aspartate aminotransferase (P11), alanine aminotransferase (P10), and uric acid levels (P5). Moreover, abnormal myocardial enzyme-related indices were found in two patients (2/13), P1 with significantly elevated creatine kinase and creatine kinase isoenzyme levels and the other (P8) with slightly increased creatine kinase isoenzyme levels. Additionally, the immunization set was performed on seven patients, and all had abnormal indicators, including IgA (3/6), IgE (3/6), IgG (4/6), and IgM (3/6). Four patients had abnormal complement levels, with reduced C3 (2/6) and C4 (2/6) levels and slightly increased C4 levels (P9). Analyses of oligoclonal bands (OCBs) and intrathecal Ig synthesis were conducted in 12 patients, two patients were positive for OCBs, and OCBs were absent in other patients (10/12) (Table 2).

### 3.5. EEG and neuroimaging findings

**Figure 4:**
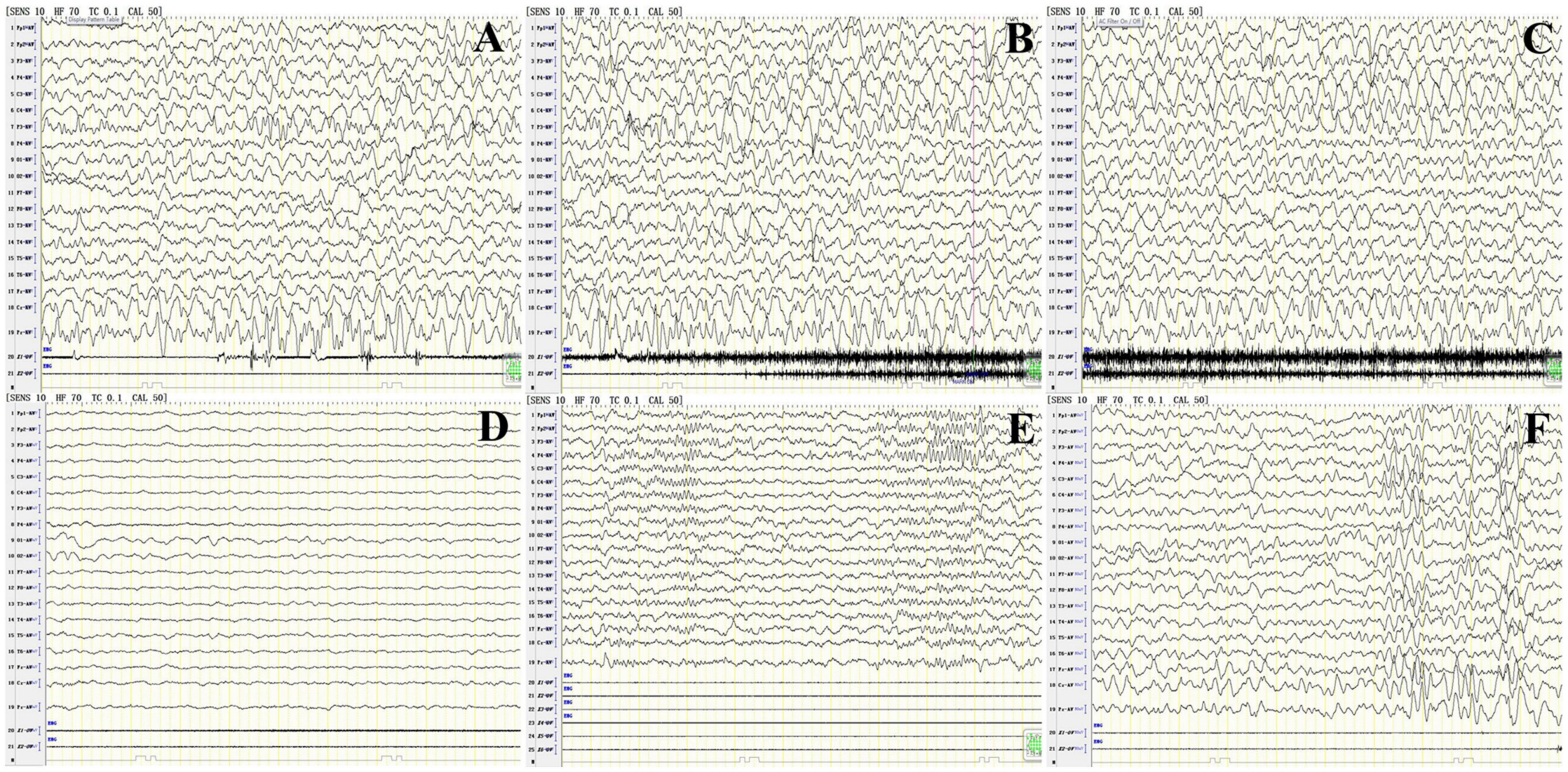
Abnormal EEG findings in patient 11 with anti-CASPR2 Ab-related encephalitis. **(A–C)** Focal episodes in a persistent state at the onset of the disease. **(D)** The background rhythm in the occipital region significantly significantly slowed 3 HZ at the onset of the disease. **(E)** Sharp wave in frontal area at the onset of the disease; **(F)** Poor rhythm modulation in the occipital area, and no epileptic wave in the full amplitude after 2 weeks of treatment. CASPR2, Contactin-Associated Protein-like 2; Ab, antibody; HZ, Hertz.

Six patients had abnormal EEGs, and P11 showed a continuous focal onset, significantly slower occipital background rhythm, and sharp waves in the frontal area, which align with the characteristics of limbic encephalitis. A review of the occipital rhythm showed poor modulation, with amplitude after 10 days and no epileptic waves in the entire range (Figure 4).

**Figure 5:**
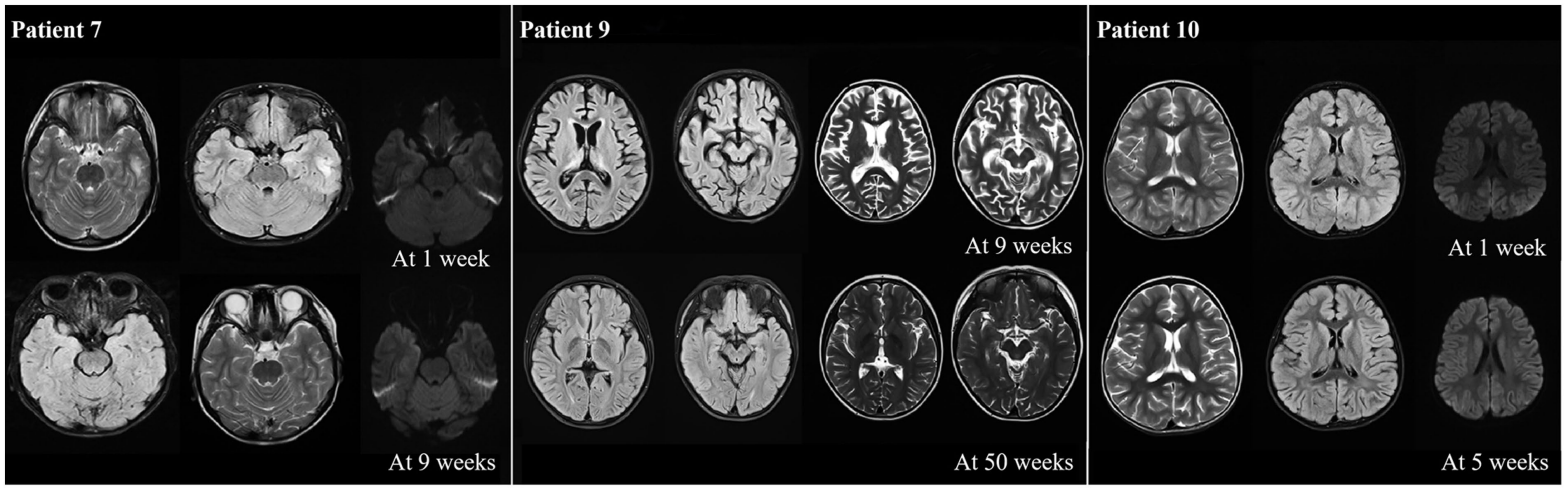
Abnormal neuroimaging findings in patients with anti-CASPR2 Ab-related encephalitis. Patient 7 with abnormal signalin bilateral frontal left temporal cortex and subcortical at one week after disease onset. Compared with onset, the left temporal cortex and subcortical signals were significantly reduced at 9 weeks after onset; the neuroimaging of P9 revealed bilateral thalamus, brain foot small sheet of abnormal signal, brain groove fissure slightly wide and deep, bilateral lateral ventricle slightly enlarged at 9 weeks from oneset, all symptoms improved at 50 weeks from oneset; the neuroimaging of P10 displayed bilateral caudate head, multiple T2 abnormalities in bilateral frontal, temporal and occipital cortex, FLAIR hyperintensity, and limited diffusion at one week after onset. After 5 weeks of treatment, all lesions decreased compared to one week of onset. CASPR2, Contactin-Associated Protein-like 2; Ab, antibody; MRI, Magnetic Resonance Imaging; FLAIR, fluid attenuated inversion recovery.

The MRI results showed that most of the patients (10/13) exhibiting different abnormalities (Table 1). An abnormal signal appeared in the bilateral frontal lobe and left temporal lobe cortex, and subcortex of P7 at one week after onset. The left temporal cortex and subcortical abnormal signal range were significantly reduced at 9 weeks after onset compared with onset. P9 presented with bilateral thalamus and right hippocampus on admission, with the possibility of viral encephalitis. The cerebral sulcus fissure was slightly broader and deeper, and the bilateral lateral ventricles were slightly enlarged at 9 weeks after the disease onset (following immunotherapy with methylprednisolone). The abnormal signal was absorbed, and the sulcus fissure and bilateral ventricle were slightly less at 50 weeks after onset than at 9 weeks after onset. P10 had multiple T2 abnormal signals in the head of the bilateral caudate nucleus, bilateral frontotemporal parietal, and occipital cortex at one week after onset. FLAIR showed hyperintensity, and limited diffusion. Most lesions in the head of the bilateral caudate nucleus, bilateral frontotemporal parietal-occipital cortex and diffusion-limited lesions were smaller at 5 weeks after onset than at one week after onset (Figure 5).

### 3.6. Treatment and outcome

Eight of the 13 patients received first-line immunotherapy strategy used both intravenous immunoglobulin (IVIG) and intravenous methylprednisolone (IVMP) pulses, and P9 also received second-line treatment with rituximab. Additionally, P10 was treated with IVIG alone. Subsequently, more than half (8/13) of the patients received oral prednisolone maintenance treatment, which lasted for 2–5 months depending on the severity of patients’ condition. P1 received an additional five times of plasma exchange and eight rounds of hematodialysis. Four patients (4/13) were treated with oxcarbazepine, carbamazepine, or clonazepam, with excellent seizures control outcomes, and have continued so far. Two of 13 patients responded well to anti-infective therapy with acyclovir or proton pump inhibitor plus amoxicillin plus clarithromycin triple therapy (Table 2).

The mRS scores of 13 patients were range 2–4 at onset, and most patients (12/13) recovered fully from motor and autonomic symptoms at two-five months after commencing immunotherapy with a mRS score range 0–1. P1 has shown partial recovery characterized by memory impairment, a significant decline in computing ability and slurred speech, and poor completion of fine movements, which with a mRS score 2. Additionally, the left limb of P1 was inflexible and the physical examination results showed that the left limb muscle strength was at level IV. Furthermore, none of the patients who recovered have had a relapse so far.

## 4. Discussion

This study included data from 13 patients with anti-CASPR2-Ab-related AE, analyzing their clinical features, abnormal EEGs, MRI changes, treatment and outcomes, aims to provide clues for clinicians to understand anti-CASPR2-Ab-related AE in children. In this study, most patients were diagnosed as AE syndrome (8/13), followed by Morvan syndrome (4/13), limbic encephalitis (3/13), epilepsy syndrome (3/13), and pain syndrome (2/13), consistent with the results in other observations (Graus et al., 2016; Boyko et al., 2020).

The review of the clinical spectrum of CASPR2 Ab syndrome revealed that the most common clinical syndrome was AE (51.5%), followed by limbic encephalitis and Morvan syndrome (Boyko et al., 2020). In addition, Syrbe et al. described the clinical phenotype of eight children with anti-CASPR2-Ab-related AE, including a combination of central symptoms such as encephalopathy, neuropsychiatric symptoms, and insomnia; autonomic symptoms such as hypertension (8/8), weight loss (6/8), sweating (6/8), arrhythmias (5/8), and gut symptoms (2/8). In our study, all patients had encephalopathy (13/13), most patients had autonomic disorders (11/13), and a few patients had pain (4/13), sleep disorders (3/13), sweating (P12), and urinary disorders (P13). Therefore, encephalopathy, neuropsychiatric symptoms, sleep disorders, weight loss, sweating are the main clinical manifestation of patients with anti-CASPR2-Ab-related AE. Due to the heterogeneity of different patients, the clinical manifestations may also be related to the age and the severity of disease.

One of the clinical manifestations caught our attention. P8 who was 2 years and one month old and had palmoplantar erythema. Although unspecified skin erythema or itching were reported in 22% (6/22) of adult patients and 16.7% (1/6) in children (Irani et al., 2012; Tan et al., 2021), palmoplantar erythema was a recurrent feature of pediatric Morvan syndrome in previous researches (Surana et al., 2019; Syrbe et al., 2020). The dehydration eczema of P8 may result from sweating, itching, and damage of autoimmune system (Steinhoff et al., 2022).

No signs of peripheral nerve involvement were found in this study, therefore, diagnosing immune-mediated encephalitis in pediatrics requires speculation. The typical adult phenotype (face-brachial dystonia seizures and hyponatremia) is absent in children, as well as symptoms of neuropathic pain and peripheral nerve overexcitation cannot be expressed or characterized well. It is difficult to identify children with anti-CASPR2-Ab-related AE, particularly in very young patients. Our results were consistent with the report of Tan et al. (2021) on anti-CASPR2-Ab-related AE in children. However, Garrido Sanabria et al. (2022) showed that in 15% of adult serum anti-CASPR2-Ab-positive patients with epilepsy, the peripheral nervous system were involved. It should be a further research to determine whether there is a difference in the condition of the peripheral nervous system between adults and children with anti-CASPR2-Ab-related AE.

In this study, the CBA tests for all patients with anti-CASPR2-Ab who were diagnosed with anti-CASPR2-Ab-related AE were positive, with the Ab titers ranging between 1:10 and 1:100. Notably, Ab titers increased as the disease progressed and decreased or disappeared as disease improved or recovered. The result further provides evidence for a pathogenic role of the anti-CASPR2-Ab in AE. In a review of CASPR2 and LGI1 autoimmunity in pediatric patients, 37.8% (14/37) patients had anti-LGI1 Abs, 37.8% (14/37) had anti-CASPR2 Abs, and 24.3% (9/37) patients were double-positive for anti-LGI1 and anti-CASPR2 Abs (Nosadini et al., 2019). In this study, we tested anti-NMDAR, anti-LGI1, anti-GAD65, anti-CASPR2, anti-AMPAR, and anti-GABAB Abs in patients with suspected of having AE. Except P1 was double positive for anti-NMDAR and anti-CASPR2 Abs, other (12/13) patients had anti-CASPR2 Ab alone. The CSF anti-NMDAR-Ab titer of P1 was 1:32, serum anti-NMDAR-Ab was negative, and serum anti-CASPR2-Ab titer was 1:10. He had an overlap syndrome, the symptoms at onset included behavioral abnormalities and limb paresthesia, which were characterized by irritability, aggressive behavior, involuntary movement, prominent neuropathic pain in the left heel, and inability to walk. During the disease, the degree of abnormal behavior and involuntary movement increased, accompanied by self-mutilation behavior and cramps at about a week after illness. Therefore, we could conclude that when the patient had double positive for AE Abs, the clinical abnormal behavior increased significantly.

The CSF routine and biochemical indices of P1 were normal, except for slightly elevated lactate dehydrogenase levels. However, different from patients who had anti-CASPR2-Ab AE alone, P1 with this overlap syndrome had more abnormal serum indicators and were the only patients with abnormal thyroid indicators. He had elevated levels of free thyroxine (FT4), creatine kinase, and myolobin. Literature survey showed that FT4 was increased under conditions of stress and immune disorders (Jaeger et al., 2021); creatine kinase and myolobin were related to seizures or syncope in children (Goksu et al., 2009). What’s more, the levels of immunoglobulin Abs such as IgE, IgG, and IgM were also increased, which indicated that immune system-associated diseases or inflammatory reactions may occur (Schou et al., 2016; Passaro et al., 2021). Blood and bone marrow cultures showed the presence of *Escherichia coli*. All abnormal indicators demonstrated that his condition was critical, and he did not show clinically satisfactory results after treatment with immunotherapy, plasma exchange, and hemodialysis. Whether these abnormalities and poor efficacy were caused by anti-CASPR2-Ab alone or by double-positive for anti-NAMDAR and anti-CASPR2 Abs needs further investigation. When patients have unusual clinical features and abnormal biochemical indicators, it is necessary to consider the possibility of overlap syndrome.

Diffuse slow waves in the EEG tracings from our patients (6/13) were observed. Although this was not a specific manifestation of anti-CASPR2-Ab-related AE, it could indicate the severity of the condition, as observed in patients with encephalitis or extensive brain damage (Baysal-Kirac et al., 2016). The condition of these six patients with obvious EEG abnormalities improved significantly after immunotherapy. Previous research demonstrated that EEG response varied follow the location of MRI lesions in patients with chronic stroke (Park et al., 2016); consistent with these research, we detected focal seizures in the patients, and the origin of the EEG activity largely matched the region with the MRI lesion. It may be conclude that EEG plays an important role in diagnosis of anti-CASPR2-Ab-related AE.

The main manifestations of brain MRI are abnormal signals with blurred edges at the lesion, T1WI isointensity or hypointensity, T2WI and FLAIR hyperintensity, and diffusion-weighted imaging (DWI) isointensity or hyperintensity. Brain MRI of patients with AE revealed that lesions could involve the hippocampus, frontal lobe, parietal lobe, temporal lobe, occipital lobe, cerebellum, and basal ganglia, with TIWI slight hypointensity, T2WI/FLAIR slight hyperintensity, and restricted-diffusion on the DWI, without obvious enhancement, with the FLAIR signal changing the most significantly. However, there are rare cases of anti-CASPR2-Ab-related AE and few imaging studies in pediatric, it’s easily to miss and misdiagnose anti-CASPR2-Ab-related AE as viral encephalitis, central nervous demyelinating disease, brain tumor and other AEs. Compared with other surveys (Syrbe et al., 2020; Devine et al., 2022), this retrospective study observed a high abnormal MRIs rate (10/13). The abnormal signals mainly manifested as unilateral or bilateral lobe or basal ganglion damage in our study. Among them, six cases (6/10) had lobar lesions, four cases (4/10) had basal ganglion lesions, and three cases (3/10) had both lobar and basal ganglion lesions simultaneously. Previous studies (Graus et al., 2016; Kelley et al., 2017) had discovered that the most common brain damage sites in patients with AE were the temporal lobes, including the amygdala and hippocampus, which can affect unilateral or bilateral sides. It is apparent that the abnormal signals of imaging findings in AE are mainly concentrated on lobar, which suggested that patients with lobar lesions should undergo a lumbar puncture to examing the CSF timely. Complete autoimmune-associated encephalitis Abs in serum and CSF should be differentiated from other CNS diseases for early diagnosis and treatment.

Immunotherapy resulted in favorable outcome with control of symptoms about AE. In this study, patients receiving immunotherapy all benefited from immunotherapy, but the intensity of immunotherapy varies depending on the patients’ clinical situation. P9 did not fully recover after receiving first-line immunotherapy with IVIG and IVMP, and received rituximab as second-line immunotherapy with good results. Although most patients (12/13) have fully recovered after receiving treatment in our results, anti-CASPR2-Ab-related AE has the potential for recurrence. The recurrence rate in Asian adults with anti-CASPR2 -Ab-related AE was 60%, especially higher among the ones treated with corticosteroids alone (Ghimire et al., 2020). However, this phenomenon has not been reported in pediatric patients, which may due to the small sample size of Ghimire et al.’s study, and no concrete conclusion can be drawn regarding the inadequacy of corticosteroids monotherapy. Consistent with the results of another study (Tan et al., 2021), none of the patients (12/13) had recurrence within one-two years after rehabilitation in this study. Moreover, unlike the high incidence of tumors in adult with anti-CASPR2-Ab-related AE, none of the tumors occurred in all children in our study. The low recurrence rate may be related to the small sample size or short follow-up time period or low rate of tumor recurrence in children, so a large sample size and long-term follow-up remains to be explored.

### 4.1. Limitations and strengths

This study had a few limitations: analysis in only in a single-center, small sample number, and low representativeness, and relatively short follow-up time may not reflect the true recurrence rate. Furthermore, the low CASPR2-Ab titer may not reflect all anti-CASPR2-Ab-related AE symptoms. However, this study provides clinical information regarding anti-CASPR2-Ab-related AE in children with the most significant number of cases domestic and international populations, summarizes the common phenotypes and symptoms of anti-CASPR2-Ab-related AE in domestic patients, and conducts frequency statistics. Simultaneously, this study provides additional information on EEG and MRI abnormalities and more biochemical indications of clinical abnormalities, providing a focus for clinical disease diagnosis.

## 5. Conclusion

Our study confirms the clinical features of anti-CASPR2-Ab-related AE in a cohort of pediatric patients. CSF and MRI abnormalities are more common in patients with anti-CASPR2-Ab-related AE. Most patients benefited from with active immunotherapy. Tumor complications in children are extremely rare. Further long-term follow-up, large sample size, and multicenter studies are required to comprehensively generalize the epidemiological characteristics, treatment guidance, and prognostic assessment of anti-CASPR2-Ab-related AE.

## Data availability statement

The datasets presented in this study can be found in online repositories. The names of the repository/repositories and accession number(s) can be found in the article/Supplementary material.

## Ethics statement

The studies involving human participants were reviewed and approved by The ethics committee of Hunan children’s hospital. Written informed consent to participate in this study was provided by the participants’ legal guardian/next of kin. Written informed consent was obtained from the individual(s), and minor(s)’ legal guardian/next of kin, for the publication of any potentially identifiable images or data included in this article.

## Author contributions

HL and QT completed the literature research and determined the research theme, ethical application, and made final submission of manuscript. WH contributed to the analysis and interpretation of data, chart sorting, and conception writing manuscript. EW completed the analysis and interpretation of laboratory results. LL completed the collection and analysis of Neuroimaging. HF, JY, QL, WQ, and DG participated in writing initial draft and revision manuscripts. All authors planned the manuscript, critically revised the initial draft, made final improvements prior to submission, contributed to the article, and approved the submitted version.

## Funding

This work was supported by National Natural Science Foundation of Hunan province (2022JJ70087) and Clinical Medical Technology Innovation Guidance Project of Hunan Province (No. 2021SK50512).

## Conflict of interest

The authors declare that the research was conducted in the absence of any commercial or financial relationships that could be construed as a potential conflict of interest.

## Publisher’s note

All claims expressed in this article are solely those of the authors and do not necessarily represent those of their affiliated organizations, or those of the publisher, the editors and the reviewers. Any product that may be evaluated in this article, or claim that may be made by its manufacturer, is not guaranteed or endorsed by the publisher.

## Abbreviations

CASPR2: Anti-contactin-associated protein 2
AE: autoimmune encephalitis
Ab: antibody
EEG: electroencephalography
NMDAR: N-methyl-D-aspartate receptor
GABABR: γ-aminobutyric acid type B receptor
LGI1: leucine-rich glial inactive protein 1
MRI: magnetic resonance images
CSF: cerebrospinal fluid
GAD65: glutamic acid decarboxylase
AMPAR: α-amino-3-hydroxy-5-methyl-4-isoxazole propionic acid receptor
CBA: cell-based assay
TBA: tissue-based assay
GABAB: γ-aminobutyric acid type B
FLAIR: fluid-attenuated inversion recovery
mRS: Modified Rankin Scale
IVIG: intravenous immunoglobulin
VGKC: voltage-gated potassium channels
DWI: diffusion-weighted imaging
