# Supplementary material for: Clinical spectrum of contactin-associated protein 2 autoimmune encephalitis in children
Source: Front Neurosci. 2023 May 18;17:1106214. doi: 10.3389/fnins.2023.1106214 (PMC10232858; doi:10.3389/fnins.2023.1106214)
Supplement: Supplementary file 1 [file Data_Sheet_1.docx]

**Figure S1 Detection of CASPR2 Ab by TBA in patient 9.**


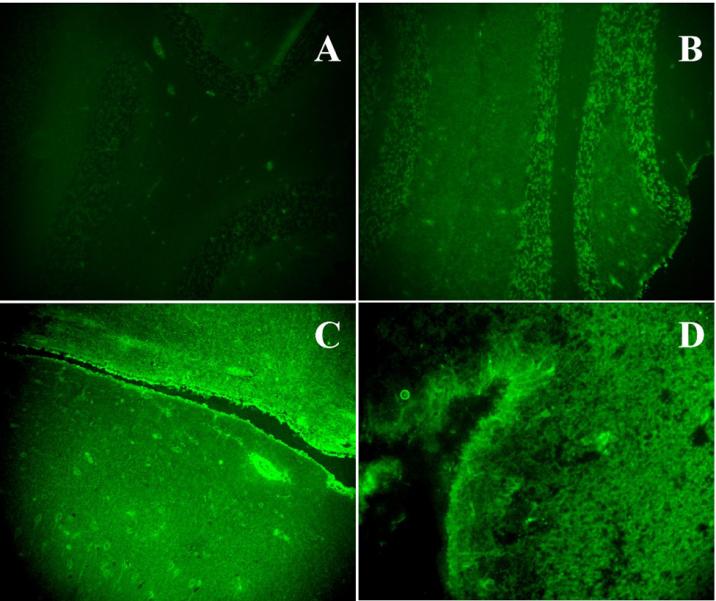


(A) Negative quality control control; (B) Positive quality control control; (C) The astrocytes and terminal processes of the pia mater also have binding（be magnified with 100X); (D) The astrocytes and terminal processes of the pia mater also have binding（be magnified with 200X).
